# Recent progress in the development of high-performance bonded magnets using rare earth–Fe compounds

**DOI:** 10.1080/14686996.2021.1944780

**Published:** 2021-09-17

**Authors:** Takashi Horikawa, Masao Yamazaki, Masashi Matsuura, Satoshi Sugimoto ‎

**Affiliations:** a Frontier Research and Development Division, Aichi Steel Corporation, Tokai, Japan; b Department of Materials Science, Graduate School of Engineering, Tohoku University, Sendai, Japan

**Keywords:** Hydrogenation disproportionation desorption recombination (HDDR), coercivity, anisotropy, oxygen, nanoparticle, 40 Optical, magnetic and electronic device materials, 203 Magnetics / Spintronics / Superconductors

## Abstract

Permanent magnets, and particularly rare earth magnets such as Nd-Fe-B, have attracted much attention because of their magnetic properties. There are two well-established techniques for obtaining sintered magnets and bonded Nd-Fe-B magnets. Powder metallurgy is used to obtain high-performance anisotropic sintered magnets. To produce bonded magnets, either melt-spinning or the hydrogenation, disproportionation, desorption, and recombination process is used to produce magnet powders, which are then mixed with binders. Since the development of Nd-Fe-B magnets, several kinds of intermetallic compounds have been reported, such as Sm_2_Fe_17_N_x_ and Sm(Fe,M)_12_ (M: Ti, V, etc.). However, it is difficult to apply a liquid-phase sintering process similar to the one used for Nd-Fe-B sintered magnets in order to produce high-performance Sm-Fe–based sintered magnets because of the low decomposition temperature of the compound and the lack of a liquid grain boundary phase like that in the Nd-Fe-B system. Therefore, bonded magnets are useful in the production of bulk magnets using these Sm-Fe-based compounds. This article reviews recent progress in our work on the development of high-performance bonded magnets using Nd_2_Fe_14_B and Sm_2_Fe_17_N_x_ compounds.

## Graphical Abstract

**Figure:**
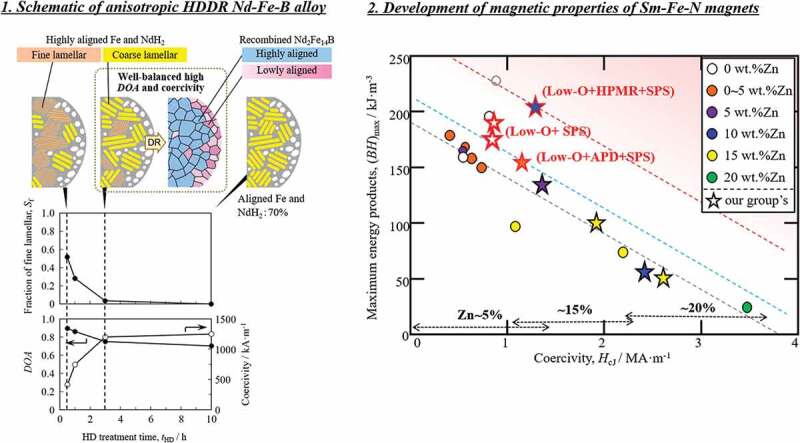

## 1. Introduction

Permanent magnets are now an essential material in many fields of technology because of their ability to provide magnetic flux and have found applications in a wide range of devices. In 1982, Nd-Fe-B magnets that use the body-centered tetragonal Nd_2_Fe_14_B phase [1] were developed by Croat et al. [2,3] using melt spinning, and by Sagawa et al. [4] using powder metallurgy techniques. The magnetic properties of the Nd_2_Fe_14_B phase are *J*_s_ = 1.61 T, μ_0_*H*_A_ = 7.2 T, and *T*_c_ = 312°C. Owing to their superior magnetic properties and low cost, Nd-Fe-B magnets have rapidly replaced Sm-Co–based magnets. Nd-Fe-B magnets with a maximum energy product (*BH*)_max_ of over 400 kJm^−3^ have been produced commercially by improving the alloy composition, ingot and powder preparation, magnetic pressing, and surface coating, and the highest (*BH*)_max_ of 474 kJm^−3^ was reported by Harimoto and Matsuura [5].

Today, there are two types of Nd-Fe-B magnets: sintered magnets and bonded magnets. Sintered magnets are produced by using powder metallurgy techniques to obtain anisotropic and fully dense sintered magnets resulting in the high (*BH*)_max_. Although (*BH*)_max_ of sintered magnets is superior to that of bonded magnets, bonded magnets have the major advantage of obtaining nearly net-shaped products and having high resistivity resulting in suppressing eddy current. Bonded magnets are fabricated by blending Nd-Fe-B magnet powders, which are widely manufactured using melt-spinning techniques or the hydrogenation, disproportionation, desorption, and recombination (HDDR) process, and binders such as thermo-elastomer or thermo-plastic resins. The Nd-Fe-B magnet powders prepared by the melt-spinning are magnetically isotropic, therefore the obtained bonded magnets are also magnetically isotropic. On the contrary, the HDDR process [6–9], which consists of a series of heat treatments in hydrogen and under vacuum, can prepare anisotropic Nd-Fe-B powders. The anisotropic Nd-Fe-B magnet powders prepared by HDDR process are used for obtaining anisotropic bonded magnets in today. To increase energy products of the bonded magnets, improving anisotropy of HDDR treated Nd-Fe-B powder is required.

Furthermore, the discovery of Nd-Fe-B magnets accelerated the search for novel and improved rare earth-Fe–based hard magnets, leading to the discovery of a number of promising magnetic materials such as ThMn_12_-type compounds [10,11], and interstitially modified Sm_2_Fe_17_ [12], ThMn_12_-type [13], and Nd_3_(Fe,Ti)_29_-type [14,15] compounds with nitrogen. The most notable of these compounds is Sm_2_Fe_17_N_x_, which offers the prospect of magnets with even better magnetic properties and a higher Curie temperature (T_c_ = 476°C) than that of Nd_2_Fe_14_B [16,17].

Sm-Fe-N powder is mass-produced by nitriding Sm_2_Fe_17_ powder, which is produced by crushing an ingot or by the reduction–diffusion method. The nitrogen is thought to occupy three 9e octahedral interstitial sites around the Sm atoms in the Sm_2_Fe_17_ compound with the rhombohedral (Th_2_Zn_17_) structure. However, this compound decomposes into SmN and Fe at temperatures above around 500–600°C. The production process for bulk magnets using this compound is therefore restricted to bonded magnets mixed with resin or low melting point metals. In addition, ThMn_12_-type compounds have attracted much attention after Hirayama et al. [18] reported that Sm(Fe,Co)_12_ thin film with the ThMn_12_-type structure exhibits higher *J*_s_ and μ_0_*H*_A_ than those of Nd_2_Fe_14_B. Thus, the ThMn_12_-type compounds can be applicable for bonded magnets for preparing bulk magnets.

This review focuses on bonded magnets, particularly high-performance R-Fe (R: rare earth) bonded magnets, and summarizes recent research related to the anisotropy mechanism of the HDDR process for high-performance Nd-Fe-B resin-bonded magnets, as well as the development of high-performance Sm_2_Fe_17_N*_x_* metal-bonded magnets using a low-melting-point alloy such as Zn.

## 2. High-performance Nd-Fe-B bonded magnets obtained by hydrogenation, disproportionation, desorption, and recombination

### 2.1. Introduction of this section

HDDR treatment is used to obtain Nd-Fe-B magnet powders with good magnetic anisotropy and high coercivity [6,8,19]. Therefore, HDDR treatment has been used to produce anisotropic bonded magnets for high-performance motors in automobiles and electronic devices [20,21]. In typical HDDR treatment, Nd_2_Fe_14_B powder prepared by hydrogen decrepitation is used as a starting material and is then decomposed into NdH_2_, Fe, and Fe_2_B by heat treatment in hydrogen. During subsequent heat treatment in a vacuum, the hydrogen is removed from NdH_2_, and the finely disproportionated Nd, Fe, and Fe_2_B recombine into fine Nd_2_Fe_14_B grains. Compared with melt-spinning, the HDDR process offers the advantage of producing anisotropic powders in which the *c*-axes of the Nd_2_Fe_14_B grains can be aligned in one direction by adding additional elements or by controlling the hydrogen pressure and temperature during the HDDR process.

It has been 30 years since the development of Nd-Fe-B powders using the HDDR process by Takeshita and Nakayama [6,7] and McGuiness et al. [8,9]. Since then, the magnetic properties of Nd-Fe-B HDDR powders have been improved. We previously reported the critical hydrogen pressure and temperature of the HDDR process and the optimum conditions for preparing anisotropic powders [22]. An HDDR process based on analogous principles, for instance the *d*-HDDR process, has also been reported [23]. In the typical treatment conditions for the *d*-HDDR process, a reduced hydrogen pressure is used for hydrogenation disproportionation (HD) treatment. This is because the degree of anisotropy is sensitive to the hydrogen pressure applied [20,22,24,25], and the best anisotropy was obtained when the hydrogen pressure during HD (*P*_HD_) was 30 kPa, and decreased with increasing *P*_HD_ [26,27]. This indicates that hydrogen pressure and temperature conditions close to the equilibrium curve of the HD and desorption recombination (DR) reactions in the pressure-temperature diagram together with the resultant reaction rate are preferred for obtaining higher anisotropy [22].

The difficulty of further improving magnetic properties using *d*-HDDR treatment is that the mechanism by which this crystallographic alignment occurs is still not fully understood despite much effort by many researchers [26,28–38]. Various arguments have been made, focusing on which phase memorizes the *c*-axis of the Nd_2_Fe_14_B phase and such components are called the ‘memory site.’

In early studies of this mechanism, including the works by Uehara et al. [28] and Tomida et al. [29,30], transmission electron microscopy (TEM) was used to observe samples after HD treatment. It was found that fine Nd_2_(Fe,M)_14_B (M = Co, Ga) particles (10–300 nm) were present within the coarse Fe or Fe_2_B matrices in the disproportionated structure. Because these fine Nd_2_Fe_14_B particles showed a crystallographic orientation close to that of the original Nd_2_Fe_14_B, it was speculated that such fine Nd_2_Fe_14_B particles might act as nucleation sites in the recombination stage, thereby forming aligned Nd_2_Fe_14_B grains. Uehara et al. indicated that these fine particles were undecomposed original Nd_2_Fe_14_B, whereas Tomida et al. demonstrated the possibility of reprecipitation. However, the coarse matrices containing the fine Nd_2_Fe_14_B particles were considered to form after a long HD treatment, and there was no orientation relationship between Fe and NdH_2_. Therefore, from a metallurgical perspective, the aligned Nd_2_Fe_14_B particles would unlikely form from disproportionated components, as Tomida et al. themselves noted [29,30]. Furthermore, the formation process of the aligned fine Nd_2_Fe_14_B particles was not fully explained. Later, Tomida et al. [32] and Gutfleisch et al. [33] reported a metastable *t*-Fe_3_B phase, which appeared in the early stage of disproportionation, before the formation of the Fe_2_B phase. This *t*-Fe_3_B phase showed a ‘one-to-one’ crystallographic relationship with the original Nd_2_Fe_14_B. As a result, the *t*-Fe_3_B phase was considered to be another candidate for the memory site. However, this model seemed to be valid for only some HDDR methods, including the so-called ‘*s*-HD’ method [33], in which the sample is heated in a vacuum and then exposed to hydrogen after reaching the desired disproportionation temperature. These candidate phases proposed as memory sites (fine Nd_2_Fe_14_B particles and/or *t*-Fe_3_B) were not observed in other HDDR methods such as *d*-HDDR, even when the resultant HDDR-treated samples showed magnetic anisotropy.

The anisotropy model with Fe_2_B as the memory site was first proposed by Gutfleisch et al. [34,35]. Among the disproportionation products (Fe, NdH_2_, Fe_2_B), only the Fe_2_B phase has the same tetragonal symmetry as the original Nd_2_Fe_14_B, so they proposed it as a candidate for the memory site. Although they found well-aligned Fe_2_B grains by TEM observation, no crystallographic orientation relationship between the memory site (Fe_2_B) and the original Nd_2_Fe_14_B was presented. Honkura et al. [26] also proposed a Fe_2_B-based model to explain the induction of anisotropy in the *d*-HDDR method. Because the degree of anisotropy in *d*-HDDR changes depending on the hydrogen pressure during HD step, they assumed that this change was due to fluctuations in the alignment of Fe_2_B, which varied according to the reaction rate of disproportionation. Sepehri-Amin et al. [37] performed TEM observation and found an orientation relationship between Fe_2_B and the original Nd_2_Fe_14_B in the early stages of the HD and DR reactions.

Meanwhile, Itakura et al. [31] indicated that the number of (Fe,Co)_2_B grains was insufficient to control the anisotropy induction of the entire powder particle and that (Fe,Co)_2_B formed later than Fe and NdH_2_. Takizawa et al. [38] reported the results of electron backscatter diffraction (EBSD) measurement of Fe_2_B after HD, showing that there were large misorientation angles of over 40° between neighboring grains, and that Fe_2_B grains had no preferential orientation. They furthermore reported the heterogeneous distribution of Fe_2_B grains in the HD-treated sample and the lack of a recombination phase at the interfaces between NdH_2_ and Fe_2_B grains in the early DR stage. These results suggested that Fe_2_B was unlikely to be a memory site.

From the beginning of research on the HDDR process, Fe and NdH_2_ have been known to crystallographically align in the lamellar structure formed after HD treatment [29,31,39–41]. However, the crystallographic orientation relationship between these two phases and the original Nd_2_Fe_14_B was not determined, despite many studies using electron diffraction patterns obtained by TEM. Recently, Takizawa et al. [38] reported that one of the <113> directions of Fe and NdH_2_ tends to align with the [001] direction of the original Nd_2_Fe_14_B. This was demonstrated for the first time by using EBSD measurements in the region that contained many crystal grains. The average preferred orientation direction was <113>, but each grain was slightly inclined from this direction. Therefore, it is reasonable that the crystallographic orientation relationship between Fe and original Nd_2_Fe_14_B was difficult to find in the above-mentioned TEM observations using a very limited part of the sample. Takizawa et al. also speculated that the Fe or Fe/NdH_2_ interface may be a possible memory site.

The importance of the presence of crystallographically aligned Fe and NdH_2_ as well as the maintenance of the crystallographic relationship between these two phases and the original Nd_2_Fe_14_B has been suggested by our group from the early stage of research on this mechanism [22,39,42]. Sugimoto et al. [22,42] indicated that the microstructure of the disproportionated sample is key to the induction of anisotropy. When the microstructure of the HD-treated sample consisted of lamellae and the crystallographic orientation relationship between Fe and NdH_2_ was maintained, higher remanence was obtained. However, when spherical NdH_2_ grains were formed and the crystallographic relationship was lost, the remanence was low. A role of the lamellar structure in the induction of anisotropy was also reported by Han et al. [43]. They showed that with increasing HD times, the degree of anisotropy decreased and the microstructure morphology of the sample changed from lamellar to spherical.

As described above, there are many models for the induction of anisotropy and memory site candidates. Among them, we believe that maintaining the crystallographic relationship of Fe, NdH_2_, and Nd_2_Fe_14_B during *d*-HDDR treatment is the most important factor in achieving higher anisotropy. Recently, we obtained experimental results that support this hypothesis [44–46]. Moreover, based on those results, we developed a novel method for preparing raw material powders for *d*-HDDR in order to improve anisotropy [47,48]. In this section, we introduce our anisotropy model [44–46] and summarize the latest results on the effect of hydrogen decryption temperature (*T*_dec_) on magnetic properties [47,48].

### 2.2. Inducing magnetic anisotropy during hydrogenation, disproportionation, desorption, and recombination treatment

#### 2.2.1. Reaction between Nd_2_Fe_14_B and hydrogen

**Figure 1.**
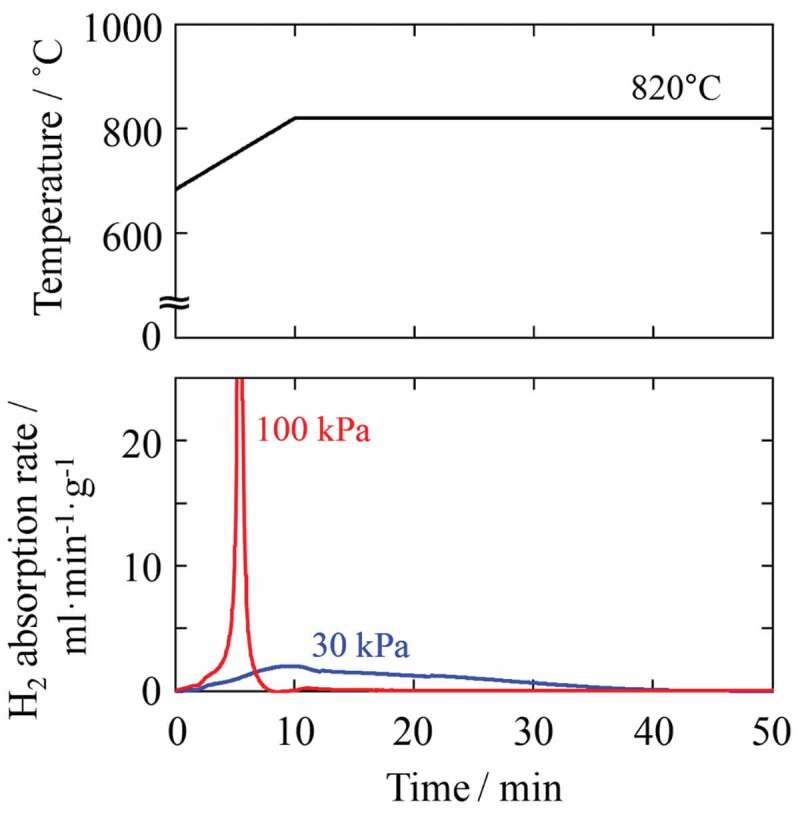
Changes in hydrogen absorption rate during HD treatment at 30 and 100 kPa. Time 0 corresponds to the start of absorption at 680°C

As described above, the degree of anisotropy varies depending particularly on *P*_HD_ and the resultant reaction rate between hydrogen and Nd_2_Fe_14_B powder particles. The driving force behind the HD or DR reaction is the change in free energy, which is small when the treatment conditions are close to the equilibrium curve of the HD and DR reactions [22]. At a typical *d*-HDDR treatment temperature of 820°C, *P*_HD_ of 30 kPa is closer to the equilibrium curve [26,27], and the change in free energy due to the HD reaction is smaller than that at 100 kPa. Therefore, the reaction rate is expected to be slow at 30 kPa, and this was confirmed by observing the hydrogen flow during HD treatment (Mass flow meter, SEF-N112, HORIBA STEC, Japan). As shown in Figure 1, when *P*_HD_ was 30 kPa, the absorption started gently and took approximately 50 min to complete. In contrast, at 100 kPa, the absorption occurred abruptly and finished in only 10 min.

**Figure 2.**
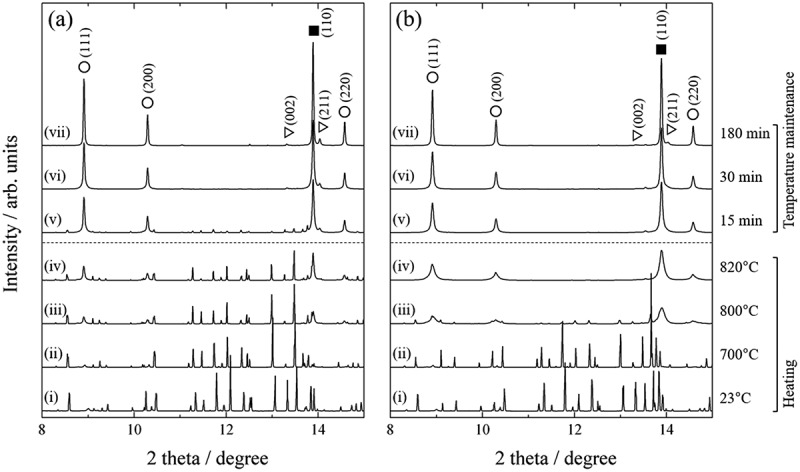
Changes in *in situ* XRD patterns observed at (a) 30 and (b) 100 kPa hydrogen pressure. (i)–(vii) show patterns acquired during heating at (i) 23°C, (ii) 700°C, (iii) 800°C, and (iv) 820°C, and patterns measured (v) 15 min, (vi) 30 min, and (vii) 180 min after reaching 820°C. Solid squares, open circles, and open triangles represent peaks attributable to Fe, NdH_2_, and Fe_2_B, respectively

The disproportionation reaction during *d*-HDDR treatment was observed directly by time-resolved *in situ* X-ray diffraction (XRD) measurement using the synchrotron radiation beam line BL02B2 at SPring-8. Details of the apparatus are described in Ref [49]. The X-ray wavelength was 0.0495786 nm. Figure 2 shows *in situ* XRD patterns during heating and HD treatment at *P*_HD_ of 30 and 100 kPa. XRD patterns were obtained by acquisition times of 2.5 and 5 min during heating and when the temperature was held at 820°C, respectively. At up to 700°C, the observed peaks as shown in Figure 2(a) were identified as those of Nd_2_Fe_14_B. The diffraction peaks of Fe, NdH_2_, and Fe_2_B phases became obvious at 800°C, and the peaks of Nd_2_Fe_14_B phase disappeared after the temperature was maintained for 30 min at 820°C. At 100 kPa (Figure 2(b)), although the peaks for Fe, NdH_2_, and Fe_2_B phases appeared at the same temperature (800°C), the Nd_2_Fe_14_B peaks almost disappeared when the temperature reached 820°C. These are a good match with the hydrogen absorption trend shown in Figure 1.

Another noteworthy point is that the crystal structure of Fe and NdH_2_ remained cubic at high temperature. We considered the possibility that the crystal structure of Fe or NdH_2_ transforms into an anisotropic structure at high temperatures owing to boron invasion or for some other reason, which could act as a memory site of anisotropy. However, because the XRD patterns acquired at 820°C were well simulated by Rietveld analyses using an isotropic cubic crystal structure for the Fe and NdH_2_ phases (*R*_wp_ < 10%), this hypothesis was rejected.

#### 2.2.2. Microstructures of hydrogenation disproportionation-treated samples

**Figure 3.**
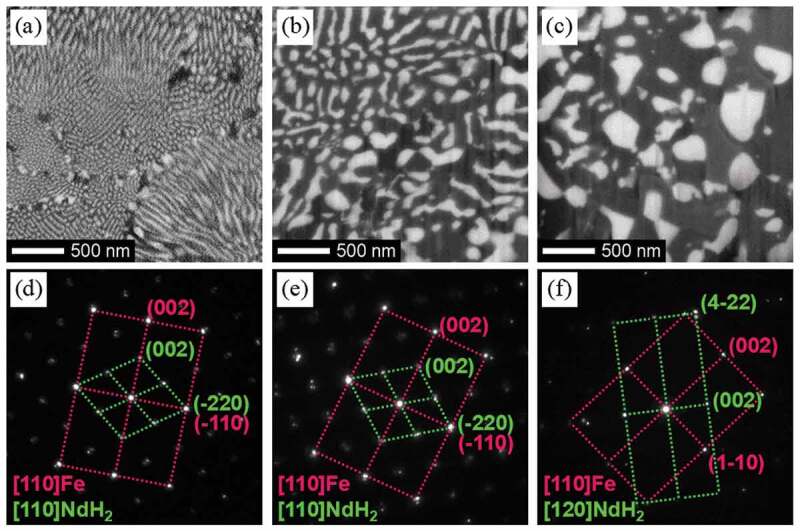
SEM-BSE images of (a) fine lamellar, (b) coarse lamellar, and (c) spherical structures. (d)–(f) show the corresponding SAD patterns

Morphological changes in the microstructure during HD treatment have been reported by many researchers [31,39,50]. In this review, we classify the microstructures observed during HD treatment into three types and their typical scanning electron microscopy–backscattered electron (SEM-BSE, JSM-7800F, JEOL, Japan) images are shown in Figure 3(a–c). The first is fine lamellar structure, which comprises rod-shaped NdH_2_ embedded in an Fe matrix (Figure 3(a)). The second is coarse lamellar structure (Figure 3(b)). Although this structure also consists of rod-shaped NdH_2_ embedded in an Fe matrix, the diameter and distance between the rods are larger and the shape is not as straight as the fine lamellar structure. Because the coarse lamellar structure is thought to result from growth of the fine lamellar structure during HD treatment, it is difficult to determine the boundary between these two structures. In this classification, if the distance between NdH_2_ rods was smaller than 50 nm, the structure was treated as fine lamellar, and if the distance was larger than 50 nm, it was treated as coarse lamellar. The third is spherical structure, in which spherical NdH_2_ grains are observed together with an Fe matrix (Figure 3(c)). This structure is usually observed after prolonged HD treatment or direct and rapid reaction between hydrogen and Nd_2_Fe_14_B phases.

As reported by many researchers [29–31,39–41,45,46], the crystallographic orientation between the Fe matrix and embedded NdH_2_ rods is the same in the fine lamellar structures. It was found that in coarse lamellar structures, the crystallographic orientation of NdH_2_ grains was also the same as that of the surrounding Fe matrix [46]. The selected area diffraction (SAD, JEM-ARM200F, JEOL, Japan) patterns observed in these two structures (Figure 3(d)) were both superposition of diffraction patterns from [110] direction of Fe and NdH_2_, and the relationship can be expressed as follows: [110]NdH_2_//[110]Fe and (−220)NdH_2_//(−110)Fe. These results indicate that the crystallographic orientation relationship between Fe and NdH_2_ is maintained even after the fine lamellar structure has grown and become coarse. However, in the spherical structures, the crystallographic orientations between Fe and NdH_2_ differ as shown in Figure 3(f). The orientation relationship between Fe and NdH_2_ was found to be different by the location in the spherical region, and no fixed relationship was observed.

**Figure 4.**
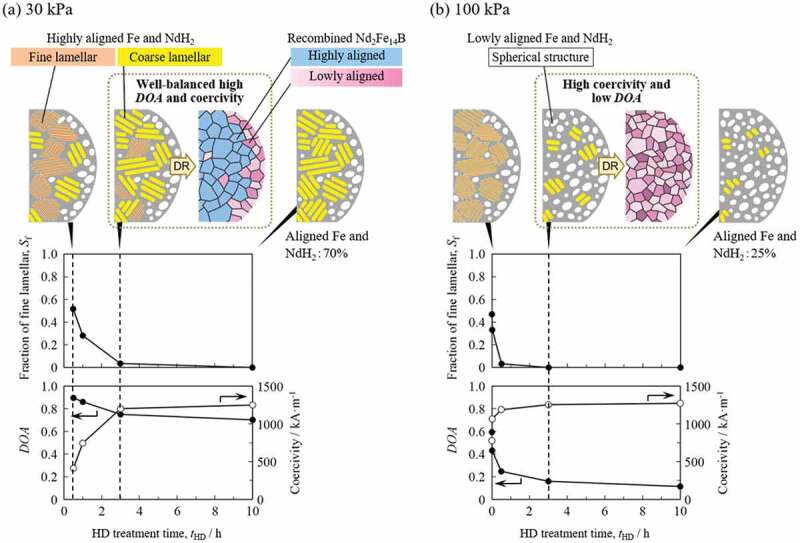
Schematic illustrations of the microstructures of HD- and DR-treated samples and *t*_HD_ dependence of area fraction of the fine lamellar structure of HD-treated samples, and DOA and coercivity of *d*-HDDR-treated samples. Orange, yellow, and white in the top illustrations represent fine lamellar, coarse lamellar, and spherical structures, respectively. Blue and pink regions after DR indicate highly and lowly aligned recombined Nd_2_Fe_14_B grains, respectively. The difference in pink color represents the deviation in crystallographic orientation. (Reproduced from [44,46], with the permission of AIP Publishing, and [45].)

Since our early study [22], we felt that the state of the HD-treated sample, and particularly the presence of crystallographic alignment of the component phases, is key to inducing anisotropy after recombination. Therefore, we rigorously investigated how the microstructure morphology and crystallographic alignment after HD treatment and the degree of anisotropy (*DOA*) after *d*-HDDR treatment depended on *P*_HD_ and HD treatment time (*t*_HD_). The *DOA* was defined as *DOA = *(*J*_r_ *− J*_r_^hard^)/*J*_r_ [43,51], where *J*_r_ and *J*_r_^hard^ are the residual magnetic polarization along the easy and hard axes of the samples after *d*-HDDR, respectively. The results are shown schematically in Figure 4. In this figure, *t*_HD_ represents the elapsed time after the temperature reached the HD temperature of 820°C. In each microstructure illustration, the left and right sides correspond to the inside and surface of the powder particle, respectively. The area fraction of the fine lamellar structure (*S*_f_) was evaluated from SEM-BSE images of each sample [44,45].

At *P*_HD_ of 30 kPa, fine lamellar structures were observed mainly in the early stage of the HD reaction (Figure 4(a)). As *t*_HD_ was increased, coarse lamellar structures were formed by the growth of fine lamellae and consequently *S*_f_ decreased. After 10 h, coarse lamellar structures became the main component, and the area fraction of coarse lamellar structures was estimated to be 0.70 using that of the crystallographically aligned Fe and NdH_2_ observed in EBSD measurements (HIKARi, TSL, Japan) [46]. When *P*_HD_ was 100 kPa, the reaction rate was higher, and the fine lamellar structure was observed in only the very early stage of the HD reaction (Figure 4(b)). After 10 h, the powder particles were composed mainly of spherical structures except for a small amount of coarse lamellae. The area fraction of the coarse lamellar was only 0.25 [46]. These results indicate that even if the fine lamellar structure disappears with increasing *t*_HD_, a large fraction of Fe and NdH_2_ remained crystallographically aligned in the coarse lamellae when *P*_HD_ was 30 kPa.

In all samples HD-treated at a *P*_HD_ of 30 kPa, spherical structures were observed near the surface or cracks in the powder particles regardless of *t*_HD_. This is because hydrogen reacts directly with sample particle and the reaction rate is high in these regions. Because the disproportionation reaction was initiated randomly throughout the entire powder particle at 100 kPa, there were no obvious microstructural differences between the inside and surface.

In summary, the morphology of microstructures after HD treatment depends on the reaction rate between hydrogen and the Nd_2_Fe_14_B phase of the powder particles, and the rate can be controlled by changing *P*_HD_. When *P*_HD_ is suitably low (30 kPa), although the microstructure near the surface or cracks becomes spherical, a large fraction of fine or coarse lamellar structures consisting of highly aligned Fe and NdH_2_ forms and remains inside the powder particles due to the optimal reaction rate. However, high *P*_HD_ (100 kPa) causes a rapid reaction, resulting in spheroidization of the microstructure in most regions of the powder particles and loss of the crystallographic orientation relationship between Fe and NdH_2_.

#### 2.2.3. Changes in degree of anisotropy and coercivity

The *t*_HD_ dependence of *DOA* was quite similar to that of *S*_f_ in both the 30 and 100 kPa treatments (Figure 4). A similar decreasing tendency was reported by Han et al. [43], who proposed that the decrease is due to disappearance of the lamellar structures after prolonged HD treatment. This idea is consistent with our anisotropy induction model [22], in which fine lamellar structures form highly oriented Nd_2_Fe_14_B. The results in Figure 4 strongly support this model. Therefore, we concluded that the first decrease in *DOA* up to *t*_HD_ of 3 h is due to a decrease in fine lamellar structures [44,45].

For the samples treated for 3 h or more, even when fine lamellar structures disappeared, a large difference was observed in *DOA* depending on *P*_HD_. This was also attributed to the difference in microstructure. As mentioned above, coarse lamellar structures remained in a large fraction (0.70) of the Fe and NdH_2_ regions at 30 kPa after 10 h. The presence of this crystallographically aligned Fe and NdH_2_ contributed to the high anisotropy. However, at 100 kPa, most of the microstructure became spherical and there was no crystallographic orientation relationship between Fe and NdH_2_, which resulted in low anisotropy [46].

The disproportionated structure is related to the grain size of the recombined Nd_2_Fe_14_B in *d*-HDDR [44]. As shown in Ref [44], the *t*_HD_ dependence of the area fraction of coarse Nd_2_Fe_14_B grains (600–1200 nm) was similar to that of *S*_f_ and *DOA* in Figure 4. Furthermore, the misorientation angle of the [001] of Nd_2_Fe_14_B was found to be small (<20°) in the coarse Nd_2_Fe_14_B grain region, but was larger (<36°) in the fine Nd_2_Fe_14_B grain region. In addition, the fine lamellar colonies (740–920 nm) and coarse grains (600–1200 nm) were similar in size. This finding suggests that fine lamellar structures form coarse Nd_2_Fe_14_B grains after recombination, and thus fine recombined Nd_2_Fe_14_B grains (200–600 nm) were thought to originate from the coarse lamellar and/or spherical structures. It is well known that coercivity strongly depends on grain size and increases with decreasing grain size. This relationship is consistent with the *t*_HD_ dependence of coercivity shown in Figure 4, where high coercivity is obtained when the disproportionated structure is composed of coarse lamellar or spherical structures and forms fine recombined Nd_2_Fe_14_B grains.

These results indicate that fine lamellar structures form coarse Nd_2_Fe_14_B grains after recombination and contribute to high *DOA*. However, coercivity decreases because of the larger grain size. In contrast, spherical structures form fine Nd_2_Fe_14_B grains. Although higher coercivity can be obtained because of the smaller grain size, *DOA* is low. From these observations, we proposed that the coarse lamellar structure is the most desirable disproportionated state in terms of both anisotropy and coercivity. For this alloy composition, well-balanced *DOA* and coercivity can be obtained at *P*_HD_ of 30 kPa and *t*_HD_ of 3 h, as shown in Figure 4, owing to the high volume fraction of the coarse lamellar structure.

### 2.3. Development of a magnet powder with higher anisotropy for enhancing the magnetic properties of Nd-Fe-B bonded magnets

#### 2.3.1. Strategy to improve anisotropy

From the anisotropy induction model discussed in section 2.2, it is expected that higher anisotropy can be obtained by reducing the fraction of spherical structures. As explained above, spherical structures usually form in regions near the surface of the particle or cracks owing to a high reaction rate with hydrogen. And these cracks are thought to form during the hydrogen decrepitation process [52] for preparing the starting powders used in *d*-HDDR treatment. When the alloy is exposed to a hydrogen atmosphere at 23°C, which is the conventional treatment temperature, both the Nd_2_Fe_14_B grains and the Nd-rich phases absorb hydrogen to form hydrides. This reaction induces a rapid volume expansion of approximately 3% and 20% in Nd_2_Fe_14_BH*_y_* and Nd-rich hydrides, respectively, thereby resulting in the formation of cracks in the Nd_2_Fe_14_BH*_y_* grains.

The hydrogen absorption limit of Nd_2_Fe_14_BH*_y_* is known to decrease with increasing temperature [53]. Therefore, the hydrogen decrepitation treatment conditions were optimized to reduce the formation of cracks.

#### 2.3.2. Dependence of amount of cracks on T_dec_

The lattice volume expansion of Nd_2_Fe_14_BH*_y_* was observed by *in situ* XRD measurements (SmartLab 9kW with Reactor X, Rigaku, Japan) and found to decrease with increasing temperature, as summarized in Table 1. A lattice volume expansion of 2.8% was observed at 23°C but decreased to 0.7% at 500°C. It is therefore expected that cracks are decreased by performing hydrogen decrepitation at higher temperature. To confirm this, the relationship between *T*_dec_ (23°C–600°C) and the crack density *D*_c_ was investigated. Here, *D*_c_ was estimated by dividing the crack length in an SEM image by the cross-sectional area of the same image.

**Table 1.** Lattice constants of Nd_2_Fe_14_B

Temperature (°C)	Hydrogen pressure (kPa)	Phase	Lattice constant (nm)		Lattice volume expansion* (%)
			*a*	*c*	
23	Vacuum	Nd_2_Fe_14_B	0.8804	1.2215	-
23	100	Nd_2_Fe_14_BH*_y_*	0.8887	1.2323	2.8
500	Vacuum	Nd_2_Fe_14_B	0.8805	1.2248	-
500	100	Nd_2_Fe_14_BH*_y_*	0.8829	1.2262	0.7

*The value indicates the degree of expansion of lattice volume at 100 kPa compared to that under vacuum at the same temperature.

**Figure 5.**
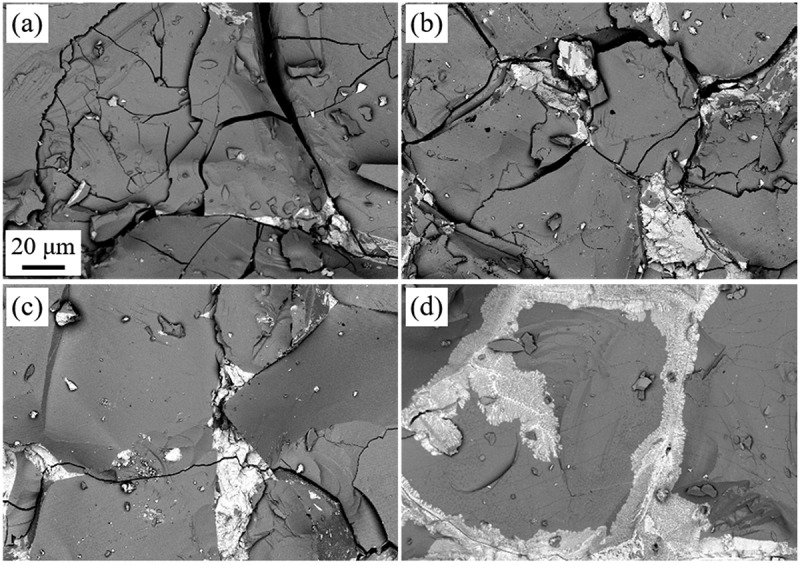
SEM-BSE images of as-crushed alloys after hydrogen decrepitation at various *T*_dec_. (a) 23°C, (b) 300°C, (c) 500°C, and (d) 600°C [47,48]

**Figure 6.**
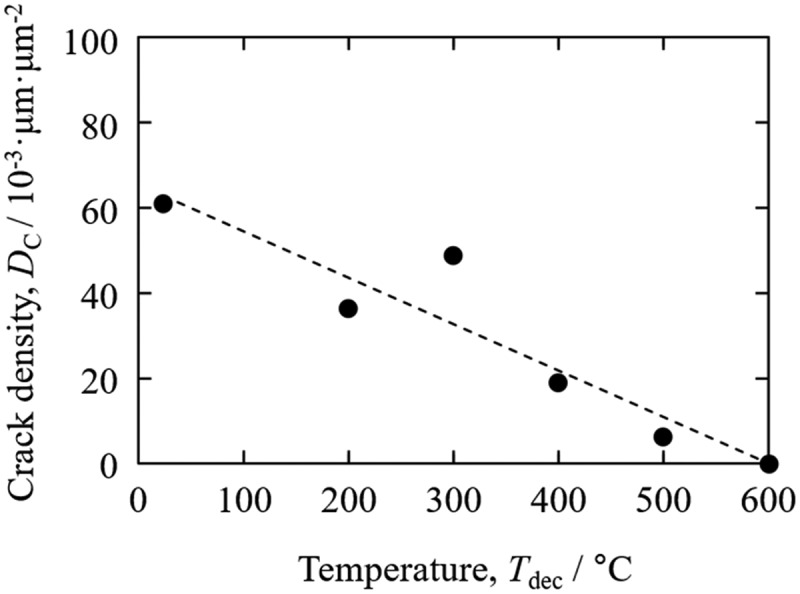
*T*_dec_ dependence of crack density in mother alloys after hydrogen decrepitation [47,48]

Figure 5 shows SEM-BSE images of as-crushed surfaces of the mother alloy after hydrogen decrepitation treatment at *T*_dec_ of 23°C–600°C. Nd_2_Fe_14_BH*_y_* grains and Nd-rich grain boundaries are observed as gray and bright contrast, respectively. The cracks in the Nd_2_Fe_14_BH*_y_* grains decreased with increasing *T*_dec_, and almost no cracks were observed at 600°C. Similarly, Nd-rich grain boundaries remained unbroken at this temperature. It was also found that disproportionation products (Fe and NdH_2_) formed near the surface of the Nd_2_Fe_14_BH*_y_* grains. The crack density *D*_c_ was evaluated using these and other corresponding SEM-BSE images and the results are shown in Figure 6. As expected, *D*_c_ decreased monotonically with increasing *T*_dec_ [47,48].

#### 2.3.3. Dependence of magnetic properties and structures on T_dec_

**Figure 7.**
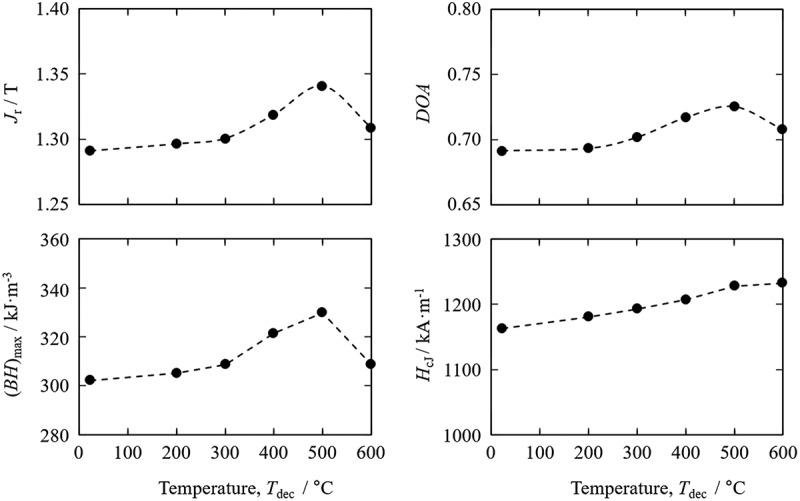
*T*_dec_ dependence of magnetic properties of *d*-HDDR-treated samples [47,48]

The cracks in the starting powder particles for *d*-HDDR treatment were effectively reduced by applying hydrogen decrepitation at high temperature. By using these starting powders, the relationship between *T*_dec_ and the magnetic properties of *d*-HDDR-treated sample powders was obtained as shown in Figure 7 [47,48]. Increases in residual magnetic polarization (*J*_r_), *DOA*, and (*BH*)_max_ was observed with increasing *T*_dec_ up to 500°C as expected from the decrease in *D*_c_ (Figure 6). From these results, the optimum *T*_dec_ was determined to be 500°C.

**Figure 8.**
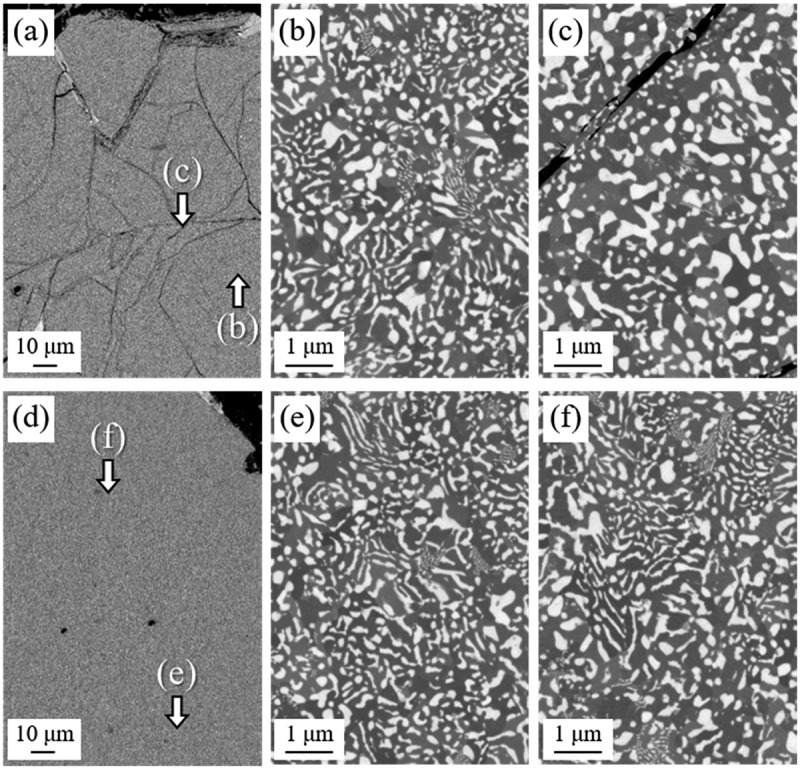
SEM-BSE images of HD-treated samples with *T*_dec_ of (a) 23°C and (d) 500°C. (b), (c), (e), and (f) show magnified views of the areas indicated in (a) and (d)

Figure 8 shows that when cracks form in Nd_2_Fe_14_B grains in the starting powder (23°C), they remain even after HD treatment (Figure 8(a)). The region far from cracks contained mainly coarse lamellar structures (Figure 8(b)); however, spherical structures were dominant in the regions near cracks (Figure 8(c)). At 500°C, powder particles were nearly free of cracks and a large amount of coarse lamellar structures was observed (Figure 8(d–f)). This observation demonstrated that the fraction of spherical structures was effectively reduced by using *T*_dec_ of 500°C.

**Figure 9.**
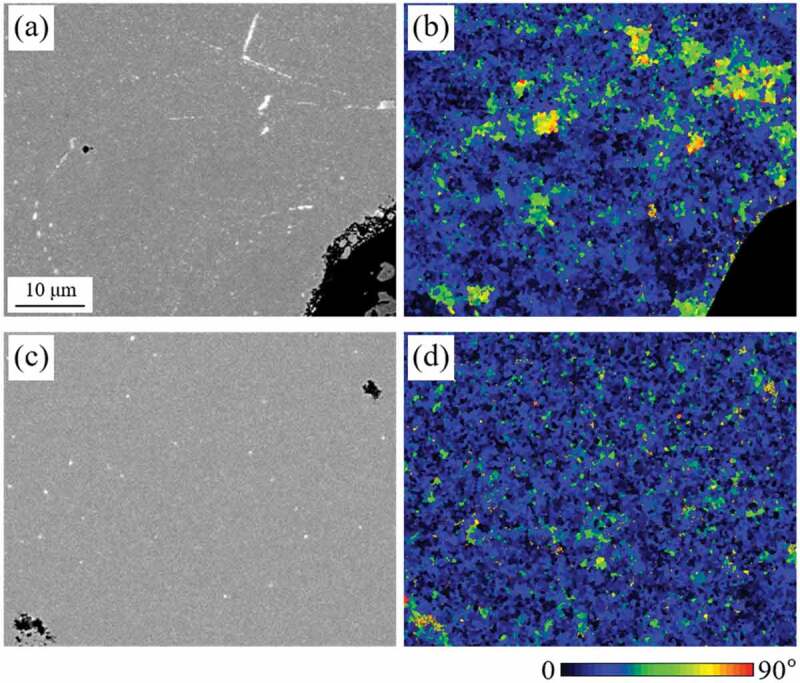
SEM-BSE images and corresponding distribution maps of the deviation angle in the Nd_2_Fe_14_B [001] direction in *d*-HDDR samples with *T*_dec_ of (a), (b) 23°C and (c), (d) 500°C, respectively. The colors in Figs. (b) and (d) indicate the amount of deviation angle [48]

Moreover, the effect of reducing cracks in the starting powders on the crystallographic alignment in the [001] direction of recombined Nd_2_Fe_14_B was confirmed by EBSD measurements. As shown in Figure 9, segregation of a bright Nd-rich phase in line and spot shapes was observed when *T*_dec_ was 23°C (Figure 9(a)), and larger deviation in [001] direction was observed near the segregation of the Nd-rich phase, as indicated by green or yellow in Figure 9(b). However, such segregation of the Nd-rich phase after HD treatment as well as regions with a large angle deviation after *d*-HDDR treatment was less obvious at 500°C because of the smaller *D*_c_ (Figure 9(c,d)) [48].

**Figure 10.**
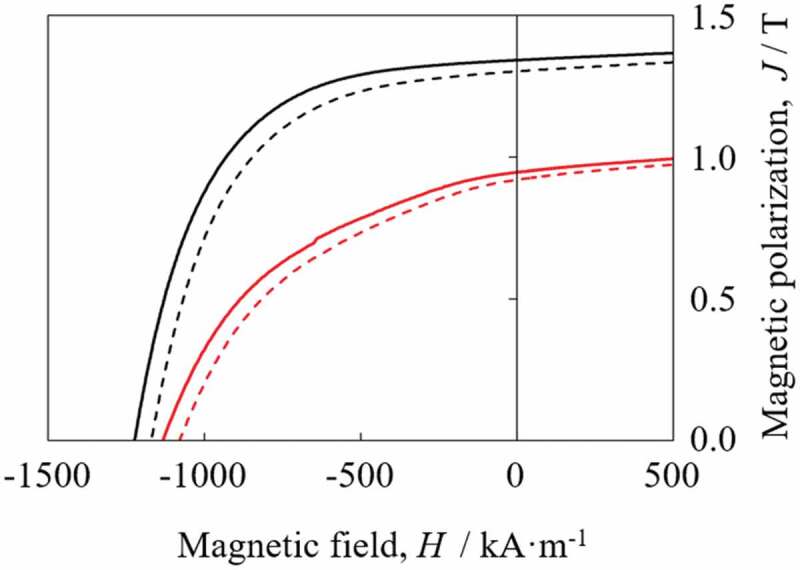
Demagnetization curves of *d*-HDDR-treated powders (black lines) and fabricated resin-bonded magnets (red lines). Dashed and solid lines represent *T*_dec_ of 23°C and 500°C, respectively [48]

Figure 10 shows demagnetization curves of powders subjected to *d*-HDDR treatment at a conventional *T*_dec_ of 23°C and an optimum *T*_dec_ of 500°C (black lines) together with those of resin-bonded magnets fabricated using the respective *d*-HDDR-treated powders (red lines) and their magnetic properties are summarized in Table 2. For the fabrication of these bonded magnets, magnet powder was mixed with approximately 3 wt% of epoxy resin binder. Then, compression molding and subsequent curing under vacuum conditions were performed. We can clearly see that *T*_dec_ of 500°C improves the magnetic properties of both the *d*-HDDR-treated powder and the resulting bonded magnet. From the structural differences shown in Figures 8 and 9, this improvement in magnetic properties was due to the increase in the fraction of coarse lamellar structures that formed highly aligned Nd_2_Fe_14_B after recombination. Improved magnetic properties of the bonded magnet can be expected from further optimization of the conditions for fabricating the bonded magnet, including the particle size of the magnetic powder, the mixing ratio of the resin, and the applied magnetic field and the pressure during the molding.

**Table 2.** *T*_dec_ dependence of magnetic properties

Sample	*T*_dec_ (°C)	*J*_r_ (T)	*DOA*	(*BH*)_max_ (kJ**·**m^−3^)	*H*_cJ_ (kA**·**m^−1^)
Powder	23	1.29	0.69	302	1160
Powder	500	1.34	0.73	330	1230
Bonded magnet	23	0.92	-	136	1100
Bonded magnet	500	0.95	-	147	1160

These results show that hydrogen decrepitation at 500°C reduces the formation of cracks in Nd_2_Fe_14_B grains in the starting powder. By using this starting powder, the fraction of spherical structures after HD treatment can be effectively reduced and the resultant angular deviations in [001] direction of recombined Nd_2_Fe_14_B are made small.

### 2.4. Summary of this section

To obtain highly aligned HDDR powders, it is important to maintain the crystallographic orientation relationship between Fe and NdH_2_ formed in both fine and coarse lamellar structures during HD treatment while suppressing the formation of spherical NdH_2_ structures. The experimental data reviewed here support this conclusion. *In situ* XRD measurement during disproportionation treatment indicated that the formation of anisotropically deformed Fe or NdH_2_ is unlikely. Suppression of crack formation by performing hydrogen decrepitation at high temperature and control of hydrogen pressure to maintain aligned structures are useful methods for producing high-performance HDDR powders and bonded magnets.

## 3. Development of high-performance Zn-bonded Sm-Fe-N magnets

### 3.1. Introduction of this section

The Th_2_Zn_17_-type Sm_2_Fe_17_N_3_ compound has a high saturation magnetic polarization (*J*_s_) of 1.54 T, a large anisotropy field (*H*_A_) of 20.6 MAm^−1^, and a high Curie temperature of 476°C [12,54]. The Sm_2_Fe_17_N_3_ phase is prepared from Sm_2_Fe_17_ phase by nitrization with nitrogen or ammonia gas at around 450°C. Generally, Sm_2_Fe_17_N_3_ powder can be obtained by a reduction-diffusion (RD) process [55–62] or by melting followed by crushing [54,63,64]. In the general RD process, Fe or Fe-O, Sm_2_O_3_, and Ca are mixed together, and the mixed powder is annealed at above the melting temperature of Ca. During annealing, Sm_2_O_3_ and Fe-O are reduced by liquid Ca, and Sm diffuses into Fe resulting in the formation of a Sm_2_Fe_17_ phase. After annealing, Sm_2_Fe_17_ powder is nitrided and washed to remove the CaO, resulting in Sm_2_Fe_17_N_3_ powder. Another process consists of preparing Sm-Fe alloy composed of Sm_2_Fe_17_ phase by melting and annealing for homogenization. The alloy is then crushed and nitrided, resulting in Sm_2_Fe_17_N_3_ powder.

Although Sm_2_Fe_17_N_3_ has good magnetic properties, high-temperature sintering cannot be applied to this alloy because of decomposition of the Sm_2_Fe_17_N_3_ phase. Since Sm_2_Fe_17_N_3_ phase decomposes to Fe and Sm-N above 500–600°C, it is necessary to develop a process for preparing bulk magnet while suppressing decomposition of this phase. One approach to preparing Sm_2_Fe_17_N_3_-based bulk magnets is to apply high pressure, such as by pressing with high pressure [65] and shock compression [66]. Saito and Kitazima [67] reported high *(BH)*_max_ of 228 kJm^−3^ by using a compression shearing method. The AIST group [65,68,69] applied a high sintering pressure to fabricate binder-free Sm-Fe-N magnets, and Takagi et al. [69] reported Sm-Fe-N bulk magnet showing good *(BH)*_max_ of 196 kJm^−3^ (=24.5 MGOe).

Another approach for obtaining bulk magnets is bonded magnets. Resin-bonded magnets have advantages such as flexibility, near net shape fabrication, and suppression of eddy currents. Today, Sm_2_Fe_17_N_3_ powder is mixed with resin binder, and resin-bonded Sm-Fe-N bulk magnets are used in motors. Our group recently reported on Mn- or Cr-diffused Sm_2_Fe_17_N_x_ core-shell powders and Sm-rich shelled Sm_2_Fe_17_N_x_ powder for application in resin-bonded magnets with high thermal stability [70–72]. To obtain bulk Sm-Fe-N magnets, attention has also fallen on metal-bonded magnets using a low-melting-point metal binder. Otani et al. [73] prepared metal-bonded Sm_2_Fe_17_N_3_ magnets using low-melting-point metals (Zn, Bi, Sn, and Al), and reported that the use of Zn as the binder improved the coercivity of Sm-Fe-N metal bonded magnets. Improved coercivity is thought to be one of the advantages of using metal binder compared with resin binder. They also pointed out that Zn_7_Fe_3_ phase, which is indexed as Γ-FeZn phase in the Fe-Zn binary alloy phase diagram, was observed in Zn-bonded Sm-Fe-N magnets after annealing. After this paper, many researchers attempted to improve the magnetic properties by investigating the phase changes of Zn-bonded Sm-Fe-N magnets [74–84]. The influence of the Γ-FeZn phase can be explained as follows: An oxidation layer is present on the surface of Sm_2_Fe_17_N_3_ powder particles [55,64,68,73,81,82] and O diffuses into the Sm_2_Fe_17_N_3_ phase during annealing. This O decomposes the Sm_2_Fe_17_N_3_ phase, and a soft magnetic α-Fe phase appears at the surface of the Sm_2_Fe_17_N_3_ phase. The α-Fe phase can act as nucleation sites for reversed magnetic domains resulting in decreased coercivity. In a recent study, our group reported the detailed microstructural changes of Zn-bonded Sm-Fe-N magnets [82]. In that study, a Zn-rich region was observed at the surface of the Sm_2_Fe_17_N_3_ phase in high-coercivity Zn-bonded magnets, and consisted of fine grains of Γ-FeZn, α-FeZn, and Sm-O. This suggests that the non-magnetic Γ-FeZn phase serves to isolate the soft magnetic α-FeZn phase from the Sm_2_Fe_17_N_3_ phase, resulting in a Zn-bonded Sm-Fe-N magnet with high coercivity.

**Figure 11.**
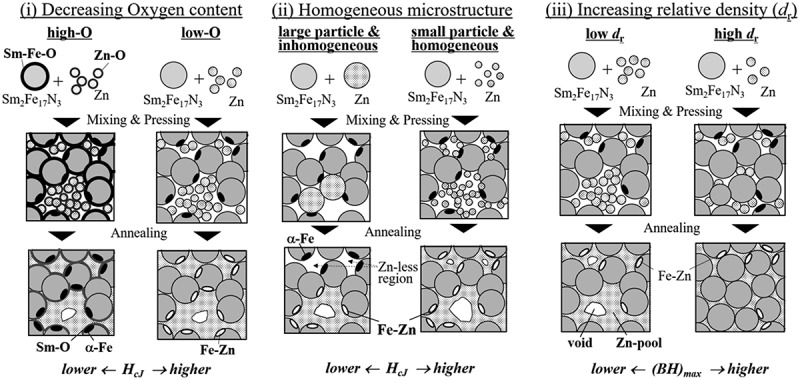
Schematics of strategy for improving magnetic properties of Zn-bonded Sm-Fe-N magnets

There are three strategies for improving the magnetic properties of Zn-bonded Sm-Fe-N magnets, as shown in Figure 11(i–iii). The first is to reduce the oxygen content. Generally, Sm-Fe-N and Zn powders have a surface oxidation layer because these powders are easily oxidized. The oxygen at the surface of the Sm-Fe-N and Zn powder can react with Sm_2_Fe_17_N_3_ phase, causing it to decompose into Sm-O and α-Fe phases because of the quite low formation energy of Sm-O. Decomposition of Sm_2_Fe_17_N_3_ phase and formation of α-Fe phase result in decreased coercivity of the Zn-bonded Sm-Fe-N magnets. Reducing the oxygen content can suppress the decomposition reaction; in other words, it can suppress the decrease in coercivity. Therefore, reducing the oxygen content in Sm_2_Fe_17_N_3_ and Zn powder can be effective for enhancing coercivity, as shown in Figure 11(i). Several papers have reported the effect of decreased oxygen content in Sm-Fe-N magnets on improved magnetic properties [65,68,69,81–84].

The second strategy is to obtain a homogeneous microstructure as shown in Figure 11(ii). In the case of inhomogeneous dispersed Zn, Zn pools can form after annealing. This effectively means that Zn does not react with α-Fe phase at the surface of Sm_2_Fe_17_N_3_ phase, and therefore the increase in coercivity is expected to be small. However, homogeneously dispersed Zn can react with α-Fe effectively resulting in a large increase in coercivity. To achieve a homogeneous microstructure, it is useful to decrease the Zn powder size. Note that reducing the powder size increases the relative surface area, making the powder more readily oxidized. Therefore, small powder size and low oxygen content need to be obtained at the same time.

The third strategy is to increase the relative density. The relative density is described by the following equation:

(1–1) $$d_{r}\;=\;\frac{d}{d_{SmFeN}\cdot W_{SmFeN}\;+\;d_{Zn}\cdot W_{Zn}}\;\times \;100$$

where *d* is the density of Zn-bonded Sm-Fe-N magnet, *d*_SmFeN_ and *d*_Zn_ are the densities of Sm_2_Fe_17_N_3_ and Zn, respectively, and *W*_SmFeN_ and *W*_Zn_ are the mass fractions of each phase. As shown in Figure 11(iii), it is clear that the *(BH)*_max_ of the magnet can be increased by increasing the density of the magnet and increasing the volume fraction of Sm_2_Fe_17_N_3_ phase because of the large volume fraction of Sm_2_Fe_17_N_3_ phase. To achieve this kind of magnet with high *(BH)*_max_, it is necessary to develop a densification process and decrease the Zn content while maintaining high coercivity. Several processes have been used to increase the relative density of Zn-bonded Sm-Fe-N magnets, including hot isostatic pressing [74], hot-rolling [78], swaging [80], and spark plasma sintering (SPS) [77,79,83,84].

Thus, to achieve high-performance Sm-Fe-N magnets, it is necessary to simultaneously obtain (i) Sm-Fe-N and Zn powders with low-oxygen content, (ii) Sm-Fe-N/Zn composite powder, and (iii) a densification process with low oxygen content. In recent years, our group has worked on developing the magnetic properties of Sm-Fe-N bulk magnets by following (i)–(iii) above. Below, we give an overview of our recent studies into the development of high-performance Zn-bonded Sm-Fe-N bulk magnets.

### 3.2. Application of arc plasma deposition and spark plasma sintering for improving magnetic properties of Zn-bonded Sm-Fe-N magnets

**Figure 12.**
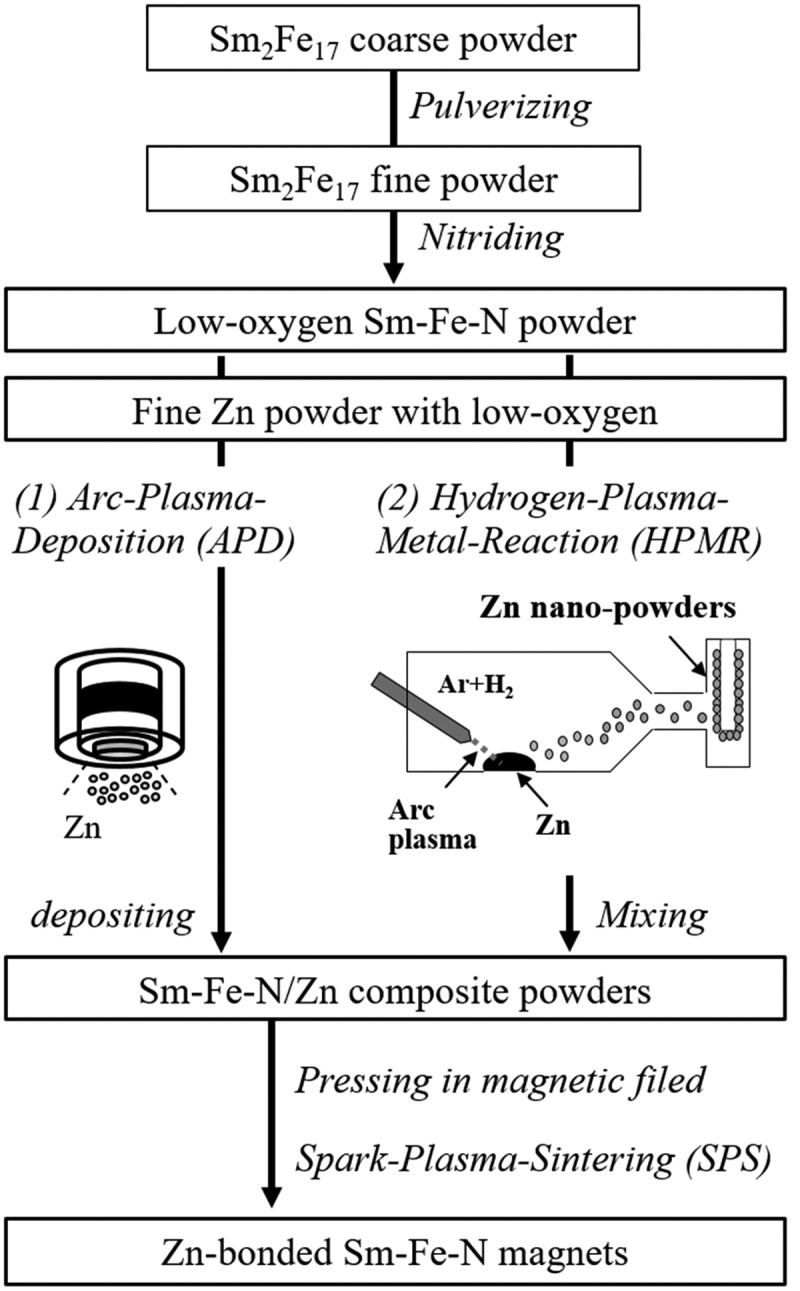
Flow chart of preparation process of Zn-bonded Sm-Fe-N magnets

**Figure 13.**
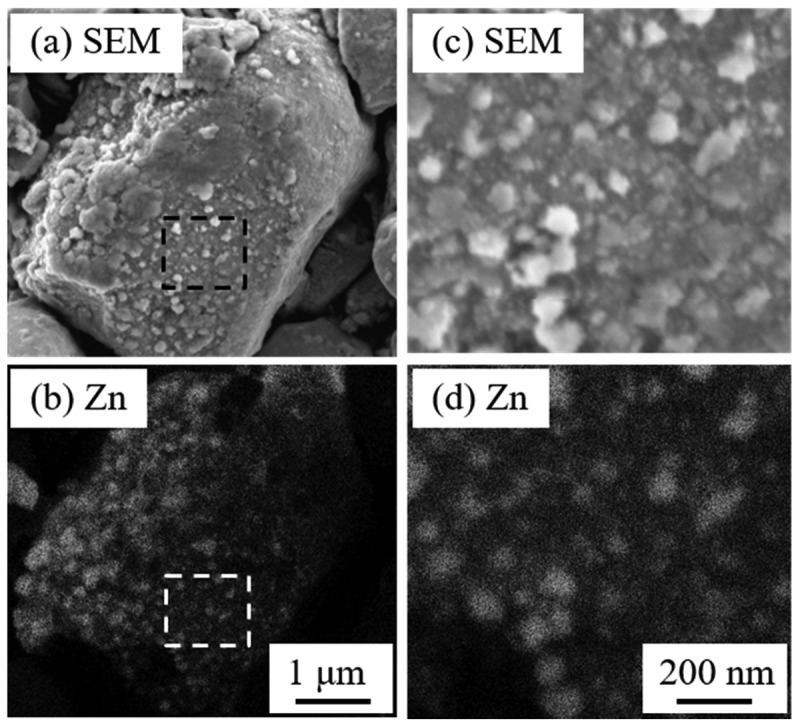
SEM and Zn mapping images of APDed Sm-Fe-N/Zn composite powders. (c) and (d) show magnified images of the squares indicated in (a) and (b) [83]. Reproduced from [83] with the permission of ELSEVIER

To increase the coercivity of Zn-bonded Sm-Fe-N magnets, it is necessary to decrease the oxygen content of the magnets. Thus, Sm-Fe-N powder with low oxygen content was prepared according to the flow chart in Figure 12. The oxygen content of the obtained Sm-Fe-N powder was approximately 0.2 wt% [83,84], which is one-third that of commercial powders. In order to achieve a homogeneous Zn dispersed microstructure, Zn-coated Sm-Fe-N powder may be suitable for obtaining high-coercivity Zn-bonded Sm-Fe-N magnets. We employed arc plasma deposition (APD) to coating the Sm-Fe-N powder with Zn to prepare Sm-Fe-N/Zn composite powder. In APD, the target is vaporized and ionized by the arc discharge, and fine particles are deposited on the particle or substrate [85]. The nanoparticle morphology and amount of deposition can be controlled by the deposition conditions such as discharge voltage and discharge count [86,87]. APD can deposit fine Zn particles with a low oxygen content on Sm-Fe-N powder because it can be performed under high-vacuum conditions. Thus, Zn was deposited on low-O Sm-Fe-N powder to obtain Sm-Fe-N/Zn composite powder. Figure 13 shows SEM and Zn mapping images of the Sm-Fe-N powder processed by APD [83]. The deposition conditions are explained in detail in a previous paper [83]. As shown in Figure 13(a,b), small particles were deposited on the surface of Sm-Fe-N powder via APD. Figure 13(c,d) are magnified SEM and elemental mapping images taken from the black and white rectangles in Figure 13(a,b), respectively. From these images, Zn particles of size several tens of nanometers were observed at the surface of the Sm-Fe-N powder. After Zn deposition, the oxygen content of the Sm-Fe-N/Zn composite powder was analyzed and found to be 0.2 wt%. Since the oxygen content of raw Sm-Fe-N was approximately 0.2 wt%, there was virtually no increase in the oxygen content as a result of the APD process. This shows that we can obtain Sm-Fe-N/Zn composite powders with low oxygen content via the APD process. Zn-bonded Sm-Fe-N magnets with good magnetic properties are expected to be obtained by using the APDed Sm-Fe-N/Zn composite powders.

**Figure 14.**
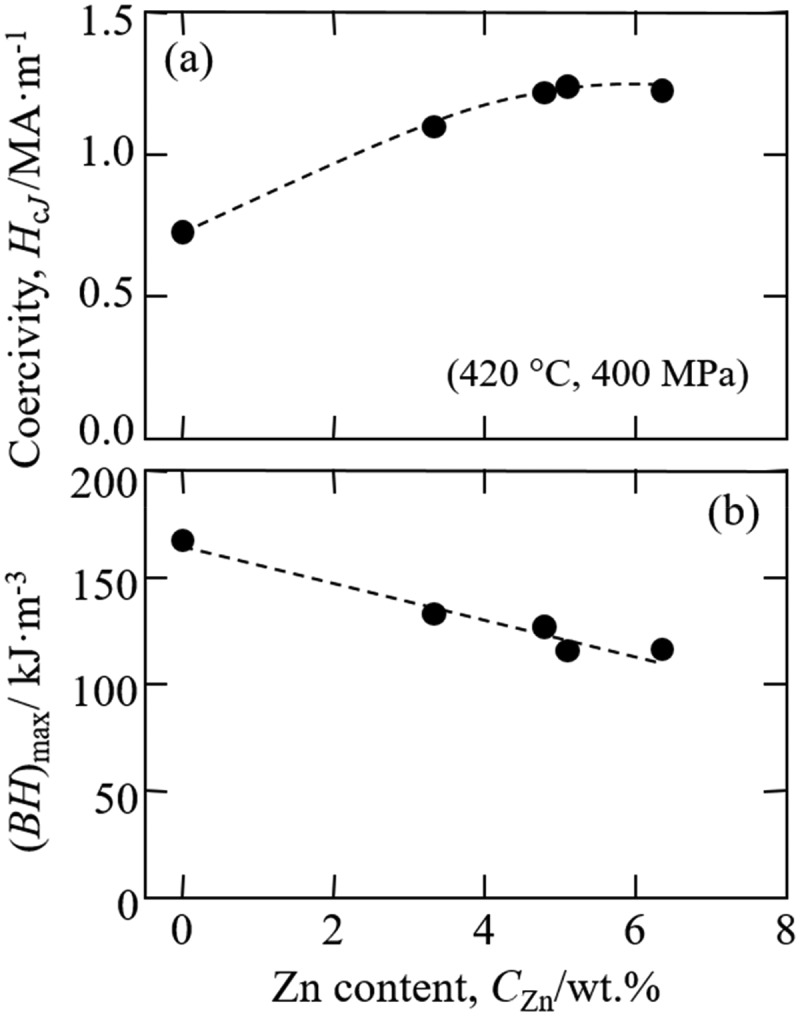
Magnetic properties of Zn-bonded Sm-Fe-N magnets prepared using Zn-deposited low-oxygen-content powders [83]. Reproduced from [83] with the permission of ELSEVIER

The low-O Sm-Fe-N/Zn composite powder was next pressed under a magnetic field and sintered by SPS to obtain a Zn-bonded Sm-Fe-N magnet. The SPS conditions were 400–750 MPa at 420°C. The oxygen content of the Zn-bonded magnets after SPS was evaluated and found to be 0.5 wt%. Although oxygen content increased slightly through the pressing and sintering process, the oxygen content of Zn-bonded magnets prepared from Sm-Fe-N/Zn composite powder processed by APD with low oxygen content was lower than that of magnets prepared using commercial Sm-Fe-N powder [83]. Figure 14 shows the magnetic properties of Zn-bonded magnets versus the Zn content. The coercivity increased with increasing Zn content. Figure 14(a) shows that a low-O magnet can maintain relatively high coercivity even if Zn-free. In Zn-bonded magnets, Zn can react with the α-Fe phase and become non-magnetic Γ-FeZn phase [73,82] resulting in magnetic isolation between Sm_2_Fe_17_N_x_ phases and increased coercivity. Therefore, the coercivity of Zn-bonded Sm-Fe-N magnets shown in Figure 14(a) increased with increasing Zn content. Figure 14(b) shows *(BH)*_max_ versus Zn content. The *(BH)*_max_ of Zn-bonded Sm-Fe-N magnets tends to increase with decreasing Zn content. As described above, the coercivity of magnets with low oxygen content can maintain relatively high values by the addition of a small amount of Zn, and therefore decreasing the amount of Zn added can increase the volume fraction of Sm_2_Fe_17_N_x_ phase, resulting in increased *(BH)*_max_.

These results indicate that *(BH)*_max_ of Zn-bonded magnets prepared using Sm-Fe-N/Zn composite powder processed by APD with low oxygen content can increase by the addition of a small amount of Zn. We next optimized the sample preparation conditions to further increase *(BH)*_max_. We optimized Zn content and sintering conditions and found that *(BH)*_max_ tends to increase with increasing sintering pressure because of the increased relative density. The relative density (*d*_r_) of the Zn-bonded (3.3 wt%-Zn) Sm-Fe-N magnet was 89% at a sintering pressure of 750 MPa, and Zn-free Sm-Fe-N magnet was also densified to *d*_r_ = 85%. These magnets exhibited the following good magnetic properties: the 3.3 wt% Zn-bonded Sm-Fe-N magnet had *(BH)*_max_ of 153 kJm^−3^ and coercivity of 1.1 MAm^−1^, and the Zn-free Sm-Fe-N magnets had *(BH)*_max_ of 179 kJm^−3^ and coercivity of 0.80 MAm^−1^ [83].

These results mean that decreasing oxygen content, increasing dispersibility of Zn, and increasing relative density were effective for enhancing *(BH)*_max_.

### 3.3. Application of Sm-Fe-N and Zn mixed powder with low oxygen content for improving magnetic properties of spark-plasma-sintered Zn-bonded Sm-Fe-N magnets

In section 3.2, we obtained good magnetic properties via a combination of APD and SPS. However, the APD process used for Zn deposition was complex. In addition, Yamaguchi et al. [88] reported that the APD process can decrease magnetization of Sm-Fe-N powder owing to the heat during deposition. Thus, we used the hydrogen plasma-metal reaction (HPMR) method to synthesize fine Zn powder with a low oxygen content. In the HPMR method, dissociated hydrogen in an arc plasma dissolves in the molten metal and enhances the vaporization of the metal [89–91]. This method makes it possible to prepare fine metal particles of sub-micrometer order sizes by controlling the hydrogen gas partial pressure and power [92–94]. We then prepared Zn fine powder via HPMR. The HPMR-Zn powder and Sm-Fe-N powder with low oxygen content were used to fabricate high-*(BH)*_max_ Zn-bonded magnets via SPS, which is shown as ‘(2)’ in Figure 12. As reported in our previous papers [81,84], it is found that the HPMR method can prepare Zn powder with sub-micrometer particle size and low oxygen content. The primary average particle and secondary average particle size of Zn were approximately 0.2 and 0.9 μm, respectively, and the oxygen content was below 700 ppm. Zn powder (10 wt%) was mixed with low-O Sm-Fe-N powder by ball-milling to obtain Sm-Fe-N/Zn mixed powder. The conditions are described in detail in our previous paper [81,84]. The oxygen content of the mixed powder was 0.27 wt%. The oxygen content of raw Sm-Fe-N and HPMR-Zn powders was approximately 0.2 and 0.07–0.08 wt.%, respectively. Therefore, the increase in the oxygen content from the mixing process was limited to only about 0.05 wt.%.

**Figure 15.**
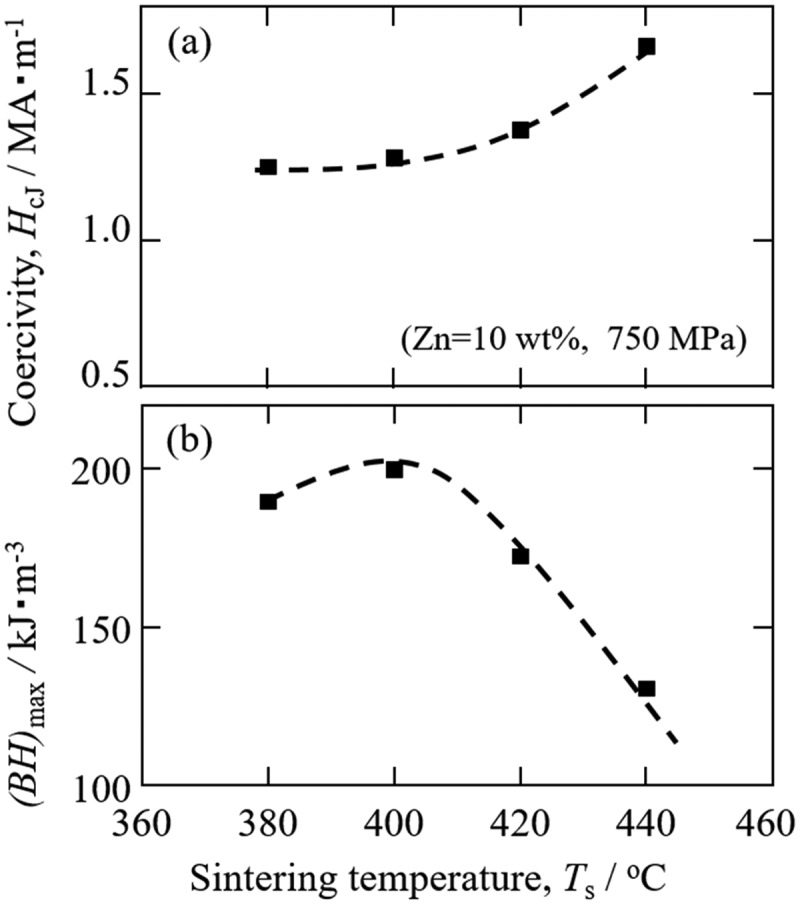
Magnetic properties of Zn-bonded Sm-Fe-N magnet prepared using low-oxygen-content Sm-Fe-N and Zn mixed powders [84]. Reproduced from [84] with the permission of the Magnetic Society of Japan

The mixed Sm-Fe-N/Zn powder was next subjected to SPS for preparation of Zn-bonded Sm-Fe-N magnets. Figure 15 shows the coercivity and *(BH)*_max_ values of 10 wt% Zn-bonded Sm-Fe-N magnets versus sintering temperature [84]. The coercivity of magnets sintered at 380°C and 400°C was around 1.2–1.3 MAm^−1^ and increased with increasing annealing temperature to 440°C. Figure 15(b) shows *(BH)*_max_ versus sintering temperature. *(BH)*_max_ increased with increasing sintering temperature to 400°C, and decreased thereafter with increasing temperature to 440°C. The highest *(BH)*_max_ of 200 kJm^−3^ (=25 MGOe) was obtained by sintering at 400°C and had a relatively high coercivity of 1.28 MAm^−1^ [84]. These are the best magnetic properties of Sm-Fe-N bulk magnets so far reported. Consequently, we successfully obtained high-*(BH)*_max_ Zn-bonded Sm-Fe-N magnets that also had relatively high coercivity.

**Figure 16.**
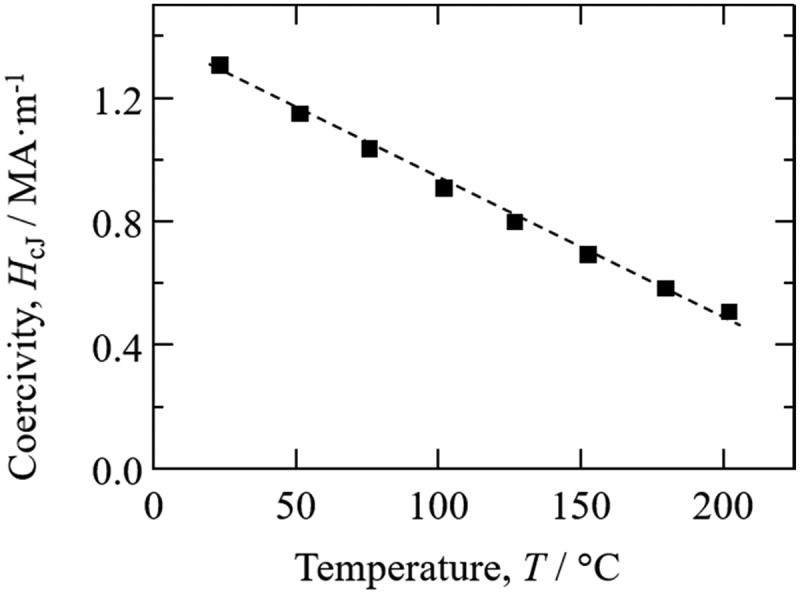
Coercivity versus temperature of Zn-bonded Sm-Fe-N magnets exhibiting high *(BH)*_max_ [84]. Reproduced from [84] with the permission of the Magnetic Society of Japan

The temperature coefficient of coercivity (α(*H*_cJ_)) is also an important parameter for magnets and was evaluated for the Zn-bonded Sm-Fe-N magnets. Figure 16 shows coercivity versus temperature for the Zn-bonded magnets over the temperature range of from 25°C to 200°C [84]. The α(*H*_cJ_) values from 25°C to 200°C for the Zn-bonded magnets were −0.34%°C^−1^. The α(*H*_cJ_) values for Zn-bonded Sm-Fe-N magnets were reported as approximately −0.36%°C^−1^ to −0.37%°C^−1^ in previous papers [95,96,97,98]. Our reported α(*H*_cJ_) values are thus thought to be better, and this improvement α(*H*_cJ_) is attributed to the decreased oxygen content of the magnets. As described in section 3.1, oxygen can diffuse into Sm-Fe-N powder and drastically decrease the coercivity of the magnets under high temperature. Therefore, decreasing the oxygen content can suppress oxygen diffusion as well as the decrease in coercivity, resulting in improved α(*H*_cJ_).

**Figure 17.**
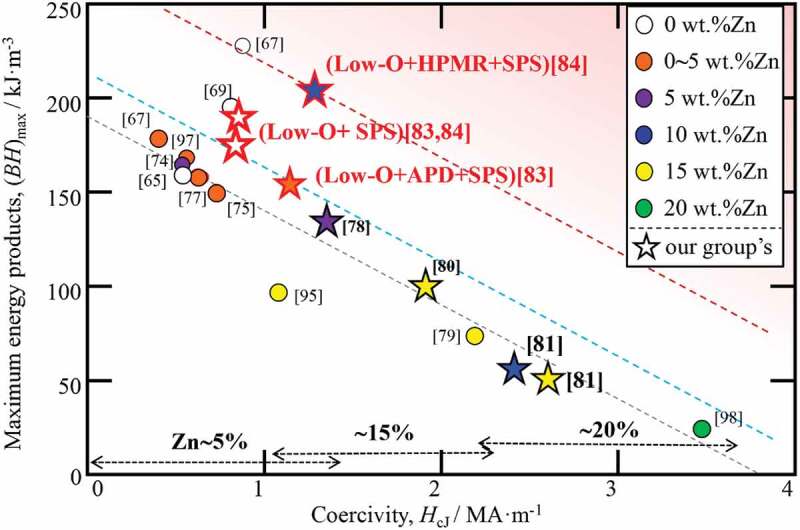
Relationship between *(BH)*_max_ and *H*_cJ_ of Zn-bonded Sm-Fe-N magnets [97,98]

Figure 17 shows the relationship between *(BH)*_max_ and *H*_cJ_ of Zn-bonded and Zn-free Sm-Fe-N magnets, as reported in previous papers. Colors indicate Zn content, and star symbol indicate values reported by our group. As can be seen in Figure 17, there is a trade-off relationship between *(BH)*_max_ and *H*_cJ_. Our recent studies [83,84], which are described in this manuscript, increased *(BH)*_max_ by a step. As described in section 3.2, we increased *(BH)*_max_ of Zn-bonded Sm-Fe-N magnets by decreasing oxygen content, increasing dispersion of Zn by APD, and increasing relative density by SPS. Good *(BH)*_max_ while maintaining a relatively high *H*_cJ_ was obtained, and the results indicate that we successfully moved the *(BH)*_max_–*H*_cJ_ line from the black dashed line to the blue dashed line in Figure 17. Furthermore, by applying fine Zn powder with low oxygen content prepared by HPMR, we successfully obtained superior magnetic properties of *(BH)*_max_ of 200 kJm^−3^ with *H*_cJ_ of 1.28 MAm^−1^. This means that we successfully moved the *(BH)*_max_–*H*_cJ_ line to the red dashed line in Figure 17.

### 3.4. Summary of this section

In the work described above, we successfully obtained high-performance Zn-bonded Sm-Fe-N magnets by (i) decreasing oxygen content through powder preparation to sintering, (ii) APD or HPMR for increasing homogeneity of microstructure, and (iii) SPS for increasing relative density. We achieved a highest *(BH)*_max_ of 200 kJm^−3^ with *H*_cJ_ and α_(HcJ)_ of 1.28 MAm^−1^ and −0.34%°C^−1^, respectively.

To further improve the magnetic properties of Zn-bonded Sm-Fe-N based magnets, it is necessary to (i) develop a powder preparation and sintering process that suppresses oxygen content to below 0.2 wt%, (ii) develop a homogeneous and thin Zn coating process for Sm-Fe-N powder, and (iii) develop a densification process for densifying at low temperature.
